# The Annual Museum

**Published:** 1894-09-01

**Authors:** 


### THE BRITISH MEDICAL ASSOCIATION AT BRISTOL.

### THE ANNUAL MUSEUM.—(*Continued*).

OPPENHEIMER, SON, AND CO. provided some of the greatest attractions in the exhibition. At Rome they were awarded the gold medal for their exhibition of palatinoids, bipalatinoids, and digestive ferments; and Dr. Heger, of Vienna, read a paper on the advantages of bipalatinoid medication, as presenting drugs in their active nascent state for absorption before destructive reaction can take place. The firm showed here a vast variety of these preparations on their handsome stall, among which their digestive ferments were prominent. These pepsines, made by the Webber process, are of various powers up to even 30,000. Their Ergole is another interesting product, which is said to contain all the active principles of ergot in a permanent solution, and freed from irritating constituents. The value of an unchanging and reliable preparation of this kind is obvious. With it they show a new hypodermic syringe, embodying all the recent improvements, and no less than 51 immediately soluble hypodermic tabloids. Among them is a one-grain sulphate of magnesia, which can be thus employed hypodermically with its usual effect. The Globe Nebulizer is a most ingenious apparatus, by which the particles of an ordinary spray are subdivided and broken up into a cloud of extreme fineness, and so introduced, without irritation, more thoroughly into the respiratory tract than would otherwise be possible. The numerous concentrated liquors, such as the Liquor euonymin and bismuthi Co., and the preparations of cascara, both as liquors and in palatinoids, are among the greatest triumphs of the pharmaceutical art. The Vibrometer, for treatment of deafness by massage, and a variety of emergency cases were also included among the numerous interesting articles they showed. Besides their beautiful stall, Messrs. Oppenheimer had an entire room containing a unique collection of ancient instruments of surgery and *ex voto* offerings unearthed from the shrines of Esculapius. The latter consist of clay models representing diseased organs, and show a knowledge of anatomy far in advance of what the ancients were believed to possess. Dr. Samboni, of Rome, kindly explained the various articles to large numbers of visitors, and the pictures, as well as the vast collection of surgical instruments from the excavations at Pompeii and elsewhere, threw a new light on the practice of ancient medicine. Catheters, specula, forceps, bistouries, and cranial elevators of various forms, with many others which have been re-invented in modern times, were displayed in large numbers, and formed such an exhibition as will be long remembered.

THOMAS CHRISTY AND CO. exhibited a great number of rare and interesting drugs and new articles. Morstadt Cachets of all kinds, and the apparatus for fitting them, Cushman's Menthol Inhaler, recommended by Lennox Browne, and their antrophors, of special value in obstinate gleet, were prominent among them. Their new vehicle for throat sprays, Benzoinol, and an atomizer, adapted for applying it are beautiful articles, and very perfectly prepared. Much interest will be taken in Analgen, a non-toxic remedy for neuralgia, rheumatism, and other pains, which was shown, together with its chemical derivatives. The N.W.K. Adeps Lanæ, and the various stages in its manufacture, made up a most instructive exhibit, with which were placed specimens of the chief preparations from it. The powder form of the Adeps Lanæ appears very valuable.

MESSRS. A. and J. WARREN, Bristol, had many of the products of Bayer and Co., europhen, salophen, &c., as well as their famous Stoddart's sea salt, used for rheumatism, sprained joints, with such success. As licensed makers of methylated spirit, which they pride themselves on manufacturing from English grain spirit only, they showed first-rate examples of their work, with the liniments in which it is employed. They exhibit a variety of soaps, and especially an inexpensive antiseptic one containing red iodide of mercury. Their ornamental one pound coppers of essence of lemon and bergamot are also noteworthy.

Fred. Stearns and Co. are remarkable for their preparations of the alkaloids of cod liver oil. They exhibited a tube of small brown crystals which contains the two chief alkaloids, morrhuine and aselline in combination. This they call Panjecorine, and claim for it a strength of two thousand times that of cod liver oil. We have tasted their Wine of Cod Liver Oil and found it most palatable, without any suggestion whatever of the oil. It may be given without a patient detecting its contents, and is certainly quite a new departure. They had also a delicious Cascara aromatic made from selected two-year-old bark, and a proteid compound of iron, their "Hæmoferrum."

The Apollinaris Company showed their Hungarian Aperient Waters; Friedrichshall, for which they are agents; and Apollinaris, the table-water of all nations, of which the sale has risen to 18 million bottles annually.

The Domen Belts' Company were noticeable for their abdominal belts, Stoop Cure, and the Varicose Circlet. This contains a curved spring and a pad filled with glycerine. It is so contrived that the circulation is not impeded, and pressure is made only upon the distended vein. It may be described an an artificial vein valve.

Messrs. J. S. Fry and Sons had one of the most artistically arranged and most popular stalls in the exhibition, which was thronged with passers-by testing their excellent cocoa. Their cocoa is extremely soluble, and their beautifully clean works, built on a gigantic scale, were inspected by many of the visitors to the Congress.

At Duncan F. Lockhart and Co.'s stall there were on view a large collection of their elegant pharmaceutical products, among which we can only notice their "Plastic" pills, the Baumol superfatted soap, and their chloroform, of world-wide reputation.

Curry and Paxton, London, Bristol and Liverpool, added to the interest of the Ophthalmic Section by their magnificent display of apparatus for eye surgery. Cooper's Optometer was prominent among the rest. With a full series of spherical and astigmatic lenses it enables the surgeon to estimate refraction in a marvellously short space of time. Quite a large collection of trial frames were on view, and the number of other new instruments showed the activity which rules in their speciality.

Godfrey and Cooke, 30, Conduit Street, Bond Street, were remarkable for the preparations of Russian Birch Tar which they exhibited. This substance, so highly esteemed by Continental dermatologists, was shown in its pure state, and in an alcoholic solution, as the Liquor Rusci Detergens, besides a skin soap, the Sapo Rusci. Their inhalers, too, for chloride of ammonium and menthol, were a noticeable feature.

Evans, Lescher, and Webb showed a bewildering assortment of drugs and preparations. They are the sole consignees for the "Montserrat" Lime Juice, such as is used in the Royal Navy. From it they manufacture delicious summer drinks—the Montserrat Squash and Limetta. They offer, too, an arrowroot of guaranteed purity from the same island. Their Savaresse's Capsules do not dissolve until they have left the stomach. This prevents much nausea and eructation in taking such drugs as copaiba. The asthma powders, and Savar's Cubeb Cigarettes, and a tasteless castor oil are highly spoken of. Their coca wine contains a fixed proportion of half a grain of the alkaloid to the ounce, and their "Saline effervescing Draught" has 1·4 per cent. of chlorate of potash with a slight excess of tartaric acid, forming an efficient and delicious compound.

At the stall of Messrs. Lorimer and Co., one of the chief novelties was Fel's germicide soap, a preparation which, containing mercuric chloride, does not, however, corrode steel. Its germicidal power is partly due to the β-Naphthol it contains, and we can testify from experience that it is very pleasant and agreeable to use. The scent, too, which is at first rather strong, becomes pleasanter and sweeter as it is used. Their other more especial preparations were the Iodised oil, which does not stain the skin like liniment; their Syrup of the hypophosphites, from an original receipt, neutral in reaction and reaching to six grains to the ounce in strength; their Coca Wine, the sample of which spoke for itself; their Lac Magnesiœ, a palatable and permanent form of fluid or rather of a suspended character; and their Oermoplastic, a well prepared ointment for eczema, made from zinc, bismuth, and boric acid.

Messrs. C. T. Hewlett and Sons presented an interesting series of new drugs. Malakin, for rheumatism; lycetol, for uric acid; chloralose, a hypnotic; gallanol, for skin disease; and gallobromol, a sedative; as well as their disinfecting fluid, which is said to be practically non-toxic, and not to corrode steel. Their flexible capsules, filled with various drugs, and the histological cabinet of one hundred microscopical specimens beautifully-stained for students, complete for £2 2s., were well worthy of a wide sale. They also showed a most extensive series of urine tests and testing apparatus, including that recently devised by Dr. Harrison for the preservation of urinary deposits.

